# Afidopyropen, a novel insecticide originating from microbial secondary extracts

**DOI:** 10.1038/s41598-022-06729-z

**Published:** 2022-02-18

**Authors:** Ryo Horikoshi, Kimihiko Goto, Masaaki Mitomi, Kazuhiko Oyama, Tomoyasu Hirose, Toshiaki Sunazuka, Satoshi Ōmura

**Affiliations:** 1 Agricultural and Veterinary Research Labs., Agricultural and Veterinary Division, Meiji Seika Pharma Co., Ltd., 760 Morooka-cho, Kohoku-ku, Yokohama, 222-8567 Japan; 2 Graduate School of Infection Control Sciences, Ōmura Satoshi Memorial Institute, Kitasato University, Tokyo, Japan

**Keywords:** Biological techniques, Drug discovery

## Abstract

Afidopyropen, a novel insecticide, is a derivative of pyripyropene A, which is produced by the filamentous fungus *Penicillium coprobium.* Afidopyropen has strong insecticidal activity against aphids and is currently used as a control agent of sucking pests worldwide. In this study, we summarized the biological properties and field efficacies of its derivatives against agricultural pests using official field trials conducted in Japan. Afidopyropen showed good residual efficacies against a variety of aphids, whiteflies and other sucking pests under field conditions. Furthermore, toxicological studies revealed its safety profiles against nontarget organisms, such as the honeybee, natural enemies and other beneficial insects, as well as mammals. Thus, afidopyropen is a next-generation agrochemical for crop protection that has a low environmental impact.

## Introduction

**Figure 1:**
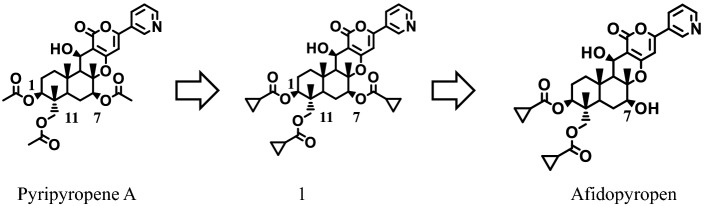
Discovery of afidopyropen.

Many pharmaceuticals and agrochemicals have been discovered from natural sources, such as extracts of plants and microbes, and they play important practical roles under field conditions. In agriculture, more than 20% of the agrochemical market consists of natural products, semi-synthetic products derived from natural compounds and biomimetic compounds^1,2^, and new agrochemical discoveries related to crop protection are still being reported^3^. Our group has focused on screening microbial extracts to discover new drugs, including pesticidal compounds^4–7^. Through the active screening of natural sources, including purified chemicals, we identified pyripyropene A (PP-A) in the Meiji natural compounds library. It was isolated as a compound that inhibited the activity of acyl-CoA:cholesterol acyltransferase^8–12^ and possessed high insecticidal activity against aphids. Aphids cause damage to crops, resulting in production losses worldwide, by feeding on plant phloem sap and vectoring a variety of viruses that cause destructive plant diseases^13^. As an insecticide, PP-A has a unique chemical structure with 3-pyridyl, α-pyrone and sesquiterpene moieties, and in aphids, exposure results in strong disorientation that ultimately leads to death^14^. Although PP-A showed good activity in laboratory assays, the residual activity in field trials was unexpectedly shorter than that of commercial standards^15^. To improve the residual efficacy in the field, we elucidated the SAR using the Kitasato’s pyripyropene derivatives library and structural optimization. After the synthesis of various derivatives, an early SAR study that focused on aphids dropping off treated leaves in laboratory assays revealed that the symptom occurred after exposure to derivatives having highly lipophilic substituents, including second lead compound **1** (Fig. 1). This compound produces a better dermal activity in foliar laboratory assays than PP-A; however, a consistent field efficacy was not achieved^16^. Therefore, as a next step, we focused on improving the oral activities of the PP-A derivatives that were ingested by aphids through the sucking of plant phloem sap, and afidopyropen emerged as a candidate insecticide. It had a log P value of 3.45 and water solubility of 25.1 mg/L. It is more hydrophilic than compound **1**, which had a log P value of 4.8 and water solubility of 0.4 mg/L^17^. A key substituent, hydroxyl, at the C7 position markedly increased not only the insecticidal activity against aphids but also the systemic activity. Afidopyropen had more than 60 times lower LC_90_ than PP-A in *Myzus persicae* (Table 1), shifting LC_90_ values from 0.45 to 0.0068 ppm, and it also exhibited excellent residual efficacy in field trials. Currently, many insecticides, such as organophosphates (OPs), carbamates, pyrethroids, neonicotinoids, ketoenols and pyrazoles, are available^18^ and are used as tools to control sucking pests. However, resistance problems to some of these insecticides have emerged in target insect pests. In addition, some insecticides are being banned or their use has been strictly limited owing to their undesirable impacts on honeybees, beneficial insects or other nontarget organisms. Therefore, new eco-friendly insecticides that aid in achieving sustainable agriculture are strongly required. Afidopyropen has been launched globally in countries such as the USA, India, China and Australia under the brand name Inscalis^®^ insecticide by BASF (Ludwigshafen, Germany), and it is expected to address the above problems while relieving negative impacts on agricultural ecosystems. Here, we summarize its insecticidal properties and effects on nontarget organisms.

**Table 1:** Insecticidal spectrum of afidopyropen against agricultural pests.

Order	Scientific name	Growth stage	LC_90_ (mg/L)		
			Afidopyropen	PP-A	Cpd. 1
Hemiptera	*Myzus persicae*	1st instar larva	0.0068	0.45	0.022
			(0.001768 < * < 0.02611)	(N.D.)	(0.01443 < * < 0.03401)
	*Aphis gossypii*	1st instar larva	0.012	0.242	0.090
			(0.009029 < * < 0.01666)	(188.5 < * < 309.9)	(0.02759 < * < 0.2906)
	*Aphis craccivora*	Mix of all stages	2.9	–	–
			(N.D.)		
	*Trialeurodes vaporariorum*	Adult	2.6	7.8	–
			(1.447 < * < 4.839)	(3.782 < * < 15.99)	
		Egg	0.13	–	–
			(0.0565 < * < 0.307)		
	*Bemisia tabaci*	Biotype B, Adult	1.5	–	–
			(1.064 < * < 2.050)		
		Biotype Q, Adult	2.1	2.6	5.8
			(N.D.)	(N.D.)	(N.D.)
	*Pseudococcus comstocki*	1st instar larva	0.3	12.1	1.3
			(0.126 < * < 0.650)	(N.D.)	(1.145 < * < 1.451)
	*Empoasca onukii*	Adult	17	–	–
			(7.365 < * < 38.76)		
	*Nilaparvata lugens*	2nd instar larva	> 100	> 100	> 100
Lepidoptera	*Plutella xylostella*	2nd instar larva	> 100	> 100	10
					(6.058 < * < 16.3)
Thysanoptera	*Frankliniella occidentalis*	1st instar larva	> 100	> 100	> 100
Diptera	*Liriomyza trifolii*	Adult	> 100	–	–
Coleoptera	*Oulema oryzae*	Adult	> 1000	–	–
Acari	*Tetranychus urticae*	Egg	> 100	> 500	> 100

–, not tested; (), 95% confidence interval; *N.D.* no data.

## Results

### Insecticidal spectra

In our study, afidopyropen showed excellent insecticidal activities against common aphid species, such as green peach (*Myzus persicae*), cotton (*Aphis gossypii*) and bean (*Aphis craccivora*), that damage a variety of vegetables, fruit trees, tea trees and ornamentals by sucking sap from sprouts and leaves. Furthermore, afidopyropen showed good activities against whiteflies (*Trialeurodes vaporariorum* and *Bemisia tabaci* Biotype Q), mealybugs (*Pseudococcus comstocki*), leafhoppers (*Empoasca onukii*) and psyllids, a hemipteran insect, and it exhibited good efficacies against these insect pests in field trials, while decreasing crop damage. These pests are common on many crops, such as cotton, beans and vegetables, and some pests have developed resistance to existing insecticides. Afidopyropen demonstrated good to excellent efficacy against multiple life stage of *T. vaporariorum*, *B. tabaci* Biotype Q and *E. onukii*. An ovicidal efficacy was not observed, but afidopyropen showed good activities against *T. vaporariorum* and *B. tabaci* Biotype Q after they hatched. It did not show insecticidal activities against Lepidoptera (*Plutella xylostella*), Thysanoptera (*Frankliniella occidentalis*), Diptera (*Liriomyza trifolii*), Coleoptera (*Oulema oryzae*) and Acari (*Tetranychus urticae*), indicating selectivity against Hemipteran pests (Table 1). Similarly, PP-A and compound **1** exhibited excellent efficacies against *M. persicae* and *A. gossypii* and moderate efficacies against *P. comstocki* and adult whiteflies. Their insecticidal spectra were the same as that of afidopyropen, except some activity observed for compound **1** against *P. xylostella*.

### Insecticidal activity against resistant insect pests

Aphids populations resistant to commercial insecticides are appearing worldwide. Some aphids are developing resistance to OP or neonicotinoids, which are widely used to control sucking pests. Therefore, we tested the efficacy of afidopyropen against resistant populations of cotton aphids collected in Japan. Afidopyropen showed excellent efficacy against both OP and neonicotinoid resistant cotton aphids collected in fields that were equivalent or superior to its efficacy against a susceptible population (Table 2). These finding demonstrate a lack of cross resistance to OP and neonicotinoids.

**Table 2:** Efficacy of a foliar afidopyropen application against cotton aphid field populations.

	LC_90_		
	Susceptible	OP resistant*	Neonicotinoids resistant*
Afidopyropen	0.012	0.008	0.002
	(0.001768 < * < 0.02611)	(N.D.)	(N.D.)
Imidacloprid	0.1638	0.2014	10.33
	(141.3 < * < 189.9)	(0.06553 < * < 0.619)	(6.705 < * < 15.92)
Acephate	3.9	> 100	87
	(0.987 < * < 15.07)		(N.D.)

*The populations collected in Japan fields.

*N.D.* no data.

**Figure 2:**
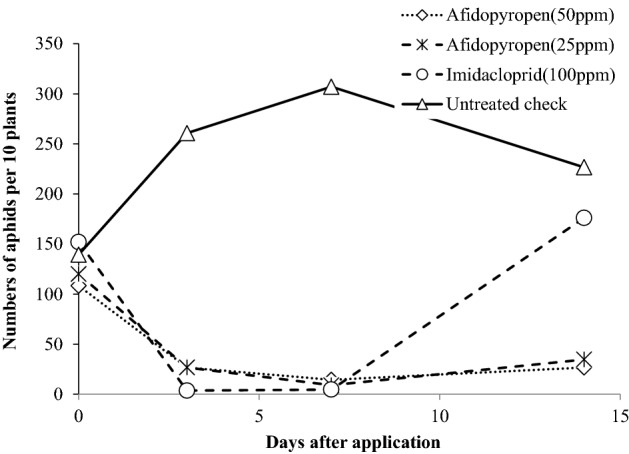
Field efficacy of foliar afidopyropen applications against cotton aphids on potato.

In addition to its excellent insecticidal efficacy against resistant populations as determined by laboratory assays, afidopyropen showed good efficacy towards populations with reduced susceptibility to commercial standards in official field trials conducted by the Japan Plant Protection Association (JPPA). In the trials, afidopyropen was used as a foliar spray at lower concentrations than imidacloprid (Fig. 2). Moreover, cotton aphids were subjected to continued selection using a foliar afidopyropen spray to assess the risk of developing afidopyropen resistance (Table 3). After breeding for 10 generations, the differences between both the LC_50_ and LC_90_ for afidopyropen were less than twofold between the F0 and F10.

**Table 3:** Efficacy of afidopyropen against the F0 (unselected) and F10 population selected by a 0.002 ppm afidopyropen spray.

Generation	LC_50_ (mg/L)	95% confidence interval	LC_90_ (mg/L)	95% Confidence interval
F0	0.00380	0.002752 < * < 0.005234	0.0184	0.009112 < * < 0.03706
F10	0.00726	0.005835 < * < 0.009035	0.0196	0.01411 < * < 0.02719

### Speed of control

**Figure 3:**
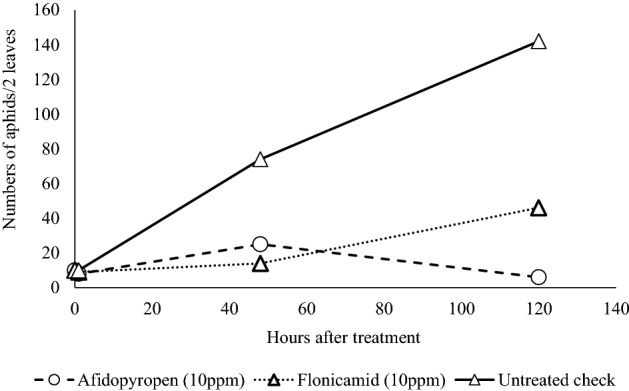
Efficacy of an afidopyropen application against adult cotton aphids after a 1-h exposure.

Aphids and whiteflies damage many crops by vectoring viruses that cause diseases. Therefore, it is essential for insecticides to quickly prevent sucking pests from transmitting viral diseases. Consequently, we evaluated the effects of a temporary exposure to a 10 ppm foliar afidopyropen spray of cucumber leaf disks on adult aphids. After a 1-h exposure, the increase in the number of aphids discontinued, and afidopyropen’s efficacy was equivalent to that of the common commercial standard flonicamid (Fig. 3).

### Systemicity of afidopyropen on crops

**Figure 4:**
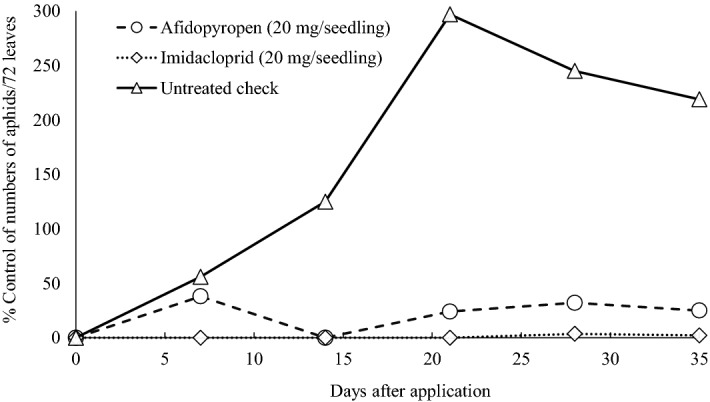
Field efficacy of afidopyropen against cotton aphids on cucumber at 7, 14, 21, 28 and 35 days after soil drenching with afidopyropen or imidacloprid. Afidopyropen or imidacloprid was placed in each transplantation hole, and the numbers of aphids naturally infesting the plants were counted at each time point.

**Figure 5:**
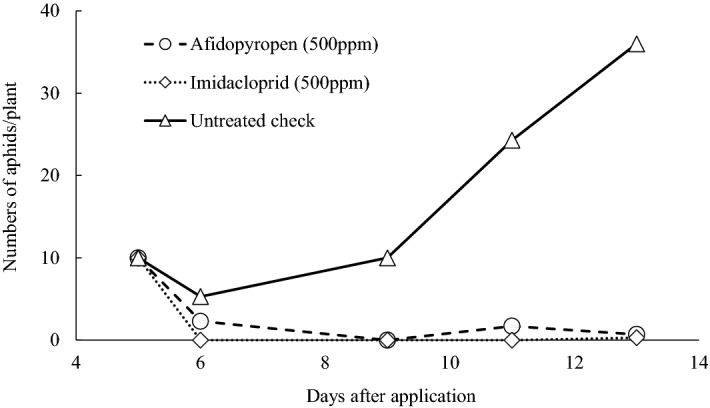
Efficacy of afidopyropen against wheat aphid by seed dipping. At 5 days after seed dipping, 10 young wheat aphid larvae were placed on each plant. At 1, 4, 6 and 8 days after infestation, the numbers of aphids were counted.

Given that aphids prefer to infest new sprouts and young undeveloped leaves, it is a desired attribute for aphid-controlling agrochemicals to penetrate from treated leaves to untreated young leaves or to untreated parts of leaves systemically within the plants. In tests to assess its systemicity from treated to untreated leaf surfaces, afidopyropen showed a good translaminar efficacy against the green peach aphid providing moderate to excellent control (Table 4). Furthermore, the upward systemicity from a treated to an untreated cucumber leaf was demonstrated with afidopyropen exhibiting over 95% aphid control on both leaves. However, the downward systemicity from a treated to an untreated downward leaf was lower with afidopyropen exhibiting 60.3% aphid control (Table 5). Because commercial standards for aphid control are frequently used in systemic applications, such as soil drenching or nursery box application in young seedlings, the root systemicity was also evaluated in a field trial. As a soil drenching agent, 20 mg afidopyropen per seedling showed a good systemicity against cotton aphids on cucumber, but the efficacy was inferior to imidacloprid (Fig. 4). Furthermore, the seed-treatment efficacy was investigated by dipping wheat seeds into the insecticide solution. Similar to soil drenching tests, afidopyropen exhibited good efficacy to wheat aphid when seeds were dipped into 500 ppm of afidopyropen solution for 6 h (Fig. 5). Although the systemicity from roots and seeds were moderate in field trials (data not shown), afidopyropen possessed good systemicity in crops and controlled aphids through some exposure routes.

**Table 4:** Translaminar efficacy of afidopyropen against green peach aphid on eggplant.

Treatment	Concentration (ppm)	Numbers of aphids	% Control
		Treated pot	
Afidopyropen	25	0	100
	12.5	32	76
	6.25	24	82
Untreated check		135	–

At 7 days after treatment of only the upside of a true leaf with each afidopyropen concentration, the number of aphids on the opposite untreated side was counted.

**Table 5:** Systemicity of topical afidopyropen application against cotton aphids on cucumber.

Systemicity	Leaf observed	Numbers of aphids		% Control
		Treated pot	Untreated pot	
Upward	2nd true leaf	1	162	99.4
	1st true leaf*	7	225	96.9
Downward	2nd true leaf*	20	369	94.6
	1st true leaf	136	343	60.3

*Treated leaves.

At 4 days after treatment of only the 1st or 2nd true leaf with 1,000 ppm afidopyropen, adult cotton aphids were placed on cucumber plants. After 8 d, the numbers of aphids were counted.

**Figure 6:**
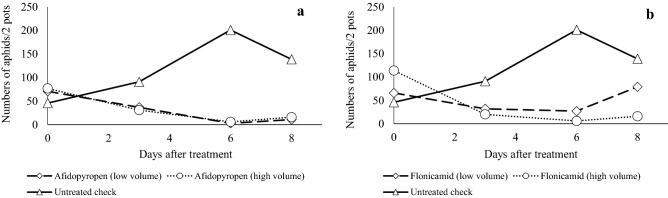
Efficacy of insecticides against wheat aphid at low (400 L/ha of 320 mg/L pesticide) and high (1280 L/ha of 100 mg/L pesticide) water volumes. (**a**) Afidopyropen; (**b**) flonicamid.

Globally, low water volumes are commonly used when spraying pesticides. To confirm its efficacy under such conditions, we evaluated the effects of spraying at a low volume (400 L/ha) of 320 mg/L afidopyropen and at a normal volume (1280 L/ha) of 100 mg/L afidopyropen. The efficacy was the same under both conditions, and the spray volume did not affect the efficacy (Fig. 6a), which was slightly better than that of flonicamid (Fig. 6b).

### Management of viral spread

**Figure 7:**
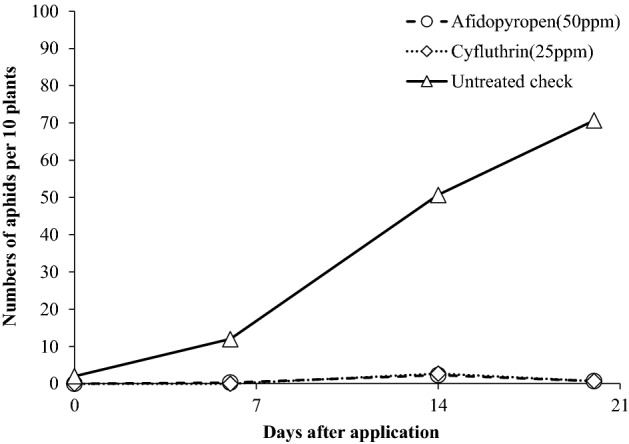
Control efficacies of weekly 4 times foliar afidopyropen applications on soybeans against greenhouse-potato aphids in a 2010 field test conducted by the Hokkaido Plant Protection Association.

Through the JPPA, we conducted field trials of an afidopyropen formulation containing 10% technical grade of active ingredient against sucking pests, and it showed good residual efficacies against aphids on vegetables and fruit trees for more than 2 weeks after a foliar application. In soybean field trials, we confirmed that 4 times 50 ppm afidopyropen foliar spray not only decreased the numbers of greenhouse potato aphids on the soybeans 6, 14 and 20 days after 1st application (Fig. 7), it also inhibited the incidence of the dwarfing disease caused by the aphid vector 62 d after 1st application. Thus, afidopyropen (50 ppm) significantly controlled soybean dwarf virus (SbDV) transmission by 27% in infested plants compared with untreated plants, as well as decreasing the numbers of aphids.

### Mode of action

Afidopyropen does not act on the nicotinic acetylcholine, GABA, glutamate, octopamine and serotonin receptors, nor on sodium channels. Furthermore, the inhibition of acetylcholine esterase, the mitochondrial electron transfer system and insect growth regulator is also not observed^19^. A recent study revealed that afidopyropen modulates the transient receptor potential vanilloid (TRPV) channels in insect chordotonal organs^20^. Chordotonal organs, which are unique to insects and crustaceans, are mechano-sensors, which are located in the joints of body segments and provide information about relative rotation of body parts. Hyperactivation and eventual silencing of TRPV channels by afidopyropen perturbs function of chordotonal organs and leads to loss of coordination, which, in turn results in inability to feed, desiccation and eventual death. Afidopyropen is classified into the 9D group of pyropene chemistry on insecticide classification by the Insecticide Resistance Action Committee^21^.

Regarding crop safety, 10% afidopyropen in water-dispersible granules has been used in a variety of Japanese field trials to confirm the insecticidal efficacy of this formulation. At an effective dose rate of 50 ppm, the foliar spray did not show any toxic effects against cereals, vegetables, tea trees, fruit trees or ornamentals.

The toxicity levels of afidopyropen against aquatic invertebrates (Table 6), honeybees (Table 7) and a variety of beneficial insects (Table 8) were low^22^. In the USA and EU, issues relating to the bee toxicity levels of existing agrochemicals are important, but a bee study using laboratory assays and semi-field trials revealed that afidopyropen has limited toxic effects. The mammalian toxicity studies revealed no serious acute, sub-acute or other toxicity issues (Table 9).

**Table 6:** Effects of afidopyropen on aquatic organisms.

Species	LC_50_ or EC_50_ or ErC_50_ (mg/L)	95% Confidence interval
*Cyprinus carpio*	18	15 < * < 23
*Daphnia magna*	8.0	5.0 < * < 15
*Pseudokirchneriella subcapitata*	> 25	N.D.

*N.D.* no data.

**Table 7:** Effects of afidopyropen on the honeybee.

Species	Application	Mortality	
*Apis mellifera in labs*	Oral	LD_50_	> 100 μg/bee
*Apis mellifera in labs*	Contact	LD_50_	> 100 μg/bee
*Apis mellifera* in semi field*	Foliar application	LD_50_	> 116.7 gai/ha

*At 3, 8, 24 and 48 h after alfalfa was treated with afidopyropen, 25 worker honeybees were released into a chamber containing collected alfalfa foliage. The deaths in each chamber were observed at 3, 24 and 48 h after release. Each test had six replicates.

**Table 8:** Effects of afidopyropen on beneficial organisms.

Species	Application	Mortality	
*Harmonia axyridis*	Contact	LD_50_	> 100 mg/L
*Chrysoperla carnea*	Contact	LD_50_	> 100 mg/L
*Aphidius colemani*	Contact	LD_50_	> 100 μg/cm^2^
*Encarsia formosa*	Contact	LD_50_	> 100 μg/cm^2^
*Orius strigicollis*	Contact	LD_50_	> 100 mg/L
*Pardosa astrigera*	Contact	LD_50_	> 100 mg/L
*Episyrphus balteatus*	Contact	LD_50_	> 100 mg/L
*Aphidoletes aphidimyza*	Contact	LD_50_	> 100 μg/cm^2^
*Anisodactylus signatus*	Contact	LD_50_	> 100 mg/L
*Eisenia foetida*	Contact	LD_50_	> 1000 mg/kg soil

The acute toxicity to all beneficial organisms was evaluated in accordance with the guidelines in the Appendix to Director General Notification, No. 12-Nousan-8147, 24 November, 2000, Agricultural Production Bureau, Ministry of Agriculture, Forestry and Fisheries of Japan or OECD No.207 guideline.

**Table 9:** Mammalian toxicity studies using afidopyropen.

Study	Effect
Acute oral toxicity to rat	LD_50_ > 2000 mg/kg
Acute dermal toxicity to rat	LD_50_ > 2000 mg/kg
Acute inhalation toxicity to rat	LD_50_ > 5.48 mg/L
Acute neurotoxicity to rat	NOEL 200 mg/kg
Sub-acute toxicity to rat in 90 days	NOEC 300 ppm
Chronic toxicity to rat in 1 year	NOEC 300 ppm
Carcinogenic toxicity to rat in 2 years	NOEC 300 ppm
2 generations reproductive toxicity to rat	NOEC 300 ppm
Developmental toxicity to rat	NOEL 30 mg/kg/day
Ames test	Negative
Skin irritation to rabbit	No irritation
Eye irritation to rabbit	Minimally irritating

## Discussion

In this study, we revealed that afidopyropen had excellent insecticidal activity against devastating piercing and sucking agricultural pests, although its insecticidal spectrum was narrow. The narrow spectrum might result from the modes of action of the TRPV modulators that help insects recognize outside mechanical stimuli and maintain proper posture and behavior in response to the stimuli, and we observed only weak activity with Cpd. 1 on *Plutella xylostella* lepidopteran larvae. The efficacy differs among the insect stages. Since hemipteran larvae are aggressively seeking foliage for feeding, the efficacy is especially high. A behavioral abnormality has also been observed in some adult hemipteran insect species, and it halts the population’s growth. Hemipteran pests damage almost all crops, genetically modified and non-genetically modified, worldwide, and even with existing insecticides, they are still not sufficiently controlled. Drug resistance and/or registration issues owing to toxicological and eco-toxicological properties of existing insecticides have negatively impacted control strategies. In particular, those of the main target pest, the aphid, with its short lifecycle and ability to quickly develop insecticide resistance. In fact, the development of resistance against major insecticides, like neonicotinoids, is remarkable^23,24^. Afidopyropen has a novel chemical scaffold and does not show cross resistance with major insecticides such as OPs, synthetic pyrethroids and neonicotinoids. In some target insects, like whitefly, even pymetrozine, which acts on TRPV, the same target protein as afidopyropen, have resistance issues develop owing to metabolic factors, but afidopyropen shows a good efficacy against pymetrozine-resistant whiteflies^25^. Moreover, our selection study using a foliar afidopyropen spray showed a low risk of resistance development. Afidopyropen is an insecticide effective by foliar spray, not only in high volume, but also in low volume applications which are globally used practices. Afidopyropen has good translaminar efficacy and systemic activity and has suitable systemic properties to control sucking pests that prefer new shoots and seedlings. In addition, afidopyropen has good control efficacies against aphids when used in soil drench and seed dip applications, as well as a good control efficacy against whitefly when used in soil drench (data not shown). However, the dose rate is higher than those of standards. Further investigations would be needed to find suitable useful scenarios in systemic uses. Although the speed of kill was slow, insects treated with afidopyropen showed abnormal behaviors in a few hours and became unable to damage plants in a short time. In fact, in some field trials, treatments stopped the development of diseases resulting from aphid-vectored viruses and very few remaining dead insects were observed because they easily fell off the treated leaves. As documented in Japanese official field trials on a variety of crops, afidopyropen has shown excellent efficacy for the control of aphids, whiteflies, leafhoppers and mealybugs.

As well, regarding consistency of efficacy across the pest spectrum overseas, afidopyropen exhibits excellent field efficacy against aphid, whitefly and the Asian citrus psyllid at low doses, 10–50 gai/ha, by BASF^26–30^, and the Food Safety Commission of Japan information indicates that it is not persistent in the environment^22^. For instance, the DT_50_ for afidopyropen is 2.7–18.6 days in soils under aerobic conditions, and 1–2 months under simulated sunlight. After application to crops, it is relatively labile with low persistence. The main metabolite, its dimer, has low acute and sub-acute toxicity levels as well.

In addition, afidopyropen shows low toxicity levels against honeybees and natural enemies, as well as against mammals. Because of environmental dynamics, afidopyropen is expected to be an eco-friendly tool of sustainable agriculture. Now that afidopyropen has been launched by BASF SE and entered into the crop protection market worldwide, it will aid in achieving sustainable agriculture. Moreover, we seek opportunities to extend this technology into unexplored crop production segments and application scenarios to improve current practices and contribute to enhanced crop productivity, including systemic uses and the treatment to control other pests and synergistic combinations.

## Methods

### General

Afidopyropen was produced and purified in accordance with our established methods^31^. The commercial insecticides imidacloprid and flonicamid were purchased from FUJIFILM Wako Pure Chemical Corporation (Tokyo, Japan). Organic solvents, chemical reagents and all the consumables were purchased from FUJIFILM Wako Pure Chemical Corporation (Osaka, Japan).

The authors confirm that we used plants commercially available in Japan in this research and all studies involving plants and plant materials were done in compliance with local and national regulations/guidelines**.**

### Insecticidal assays

#### Laboratory insecticidal tests against agricultural pests

Afidopyropen was evaluated using each target insect pest on leaf disks or potted plants in accordance with our previous report^32^. The test conditions are summarized in the Supplementary Information section. Resistant populations and populations present on 0.002 ppm afidopyropen (50% acetone/distilled water containing 0.05% Tween 20) solution-treated cucumber leaves were also tested with cotton aphids using the same leaf disk assays. Cabbage (Kinkei 201, purchased from Sakata), cucumber (Suyo, purchased from Sakata), fava bean (Funaokaissun, purchased from Takayama seed), kidney bean (Celina, purchased from Takii), wheat (Nourin 61, seeds collected inhouse), rice (Jikkoku, seeds collected inhouse) or tea (Yabukita planted inhouse) plants were used for each test, which are available in Japan. LC_90_ was calculated in a probit method with ECOTOX v3 software.

#### Speed of control assay

Adult cotton aphids were exposed to glass dishes treated with each insecticide after drying for 1 h. Then, five treated adults were placed on a cucumber (Suyo) leaf disks. At 1, 48, 120 and 168 h after infestation, the numbers of live adults and larvae were counted. The test was conducted with two replicates.

#### Translaminar assay

At 7 days after treating only the upside of an eggplant (Senryou 2, purchased from Takii) true leaf with each afidopyropen solution concentration, four adults of green peach aphids were placed on the opposite untreated leaves of the 2.8 cm diameter of leaves cut from the treated eggplants. The number of aphids on the opposite untreated sides was counted. The test was conducted with three replicates.

#### Systemic activity from treated leaves

At 4 days after treating only the 1st or 2nd cucumber (Suyo) true leaf with 1000-ppm afidopyropen, three adult cotton aphids were placed on the leaves. The numbers of aphids on both the treated and untreated upward/downward leaves were counted after 8 days. The test was conducted with two replicates.

#### Soil drenching efficacy

When transplanting 3-week-old cucumber (Suyo) seedlings, afidopyropen formulation or a commercial insecticide imidacloprid was applied into each transplantation hole. At 7, 14, 21, 28 and 35 days after transplantation, the numbers of aphids on 72 leaves from 12 plants were counted. The test was conducted in triplicate, with 12 plants per replicate.

#### Seed dipping efficacy

The wheat (Nourin 61) seeds were dipped in a water-based dilution of 5% wettable afidopyropen powder for 6 h. After the treatment, seeds were allowed to put up shoots for 72 h. Then, the seeds were transplanted. At 2 days after transplantation, 10 adult wheat aphids were placed onto the seedlings. At 1, 4, 6 and 8 days after infestation, the number of aphids was counted in each plot. This test was conducted in triplicate.

#### Efficacy of foliar applications against cotton aphid (*A. gossypii*) on potato

A JPPA field trial was conducted in 2011 using 5-week-old potato (Nishiyutaka, available in Japan) plants. A solution of afidopyropen dissolved in water was applied at 2000 L/ha to potatoes naturally infested with the cotton aphid. At 3, 7 and 14 days after application, the number of aphids was counted on 10 plants in each plot. Each plot was tested in triplicate.

1 $${\text{Corrected density index }} = \,[ {( {\text{number of aphids on treated plant at X days after application}} )/( {\text{number of aphids on treated plant before application}} )} ] \, \times \,[ {( {\text{number of aphids on untreated plant before application}} )/( {\text{number of aphids on untreated plant at X days after application}} )} ] \, \times 100.$$

Then, compared with the untreated plants, the control rate was calculated as follows:

2 $${\text{control rate }} (\%) = 100 - ( {\text{corrected density index}} ).$$

#### Management of viral spread

A field trial was conducted in 2010 by the Hokkaido Plant Protection Association using approximately 2-month-old soybean (Toyomusume, available in Japan) plants. A solution of afidopyropen dissolved in water was applied at 150 L/ha to soybeans naturally infested with the glasshouse potato aphid *Aulacorthum solani*. At 3, 7 and 13 days after application, the number of aphids was counted on 10 plants in each plot. Furthermore, the number of plants infested by SbDV was counted 13 days after application. Each test was conducted in triplicate. The control rate (%) for infested aphids was calculated using Eq. (2), and SbDV infection (%) and control rate (%), compared with untreated plants, was calculated using the following formulae:

3 $${\text{SbDV rate }} ( \% ) =( {\text{number of infected plants on treated plant at 13 days after application}} )/( {\text{number of infected untreated plants atX days after application}} ) \, \times { 1}00.$$

and

4 $${\text{control rate }}( \% ) = 100 - ( {\text{infected rate of SbDV}} ).$$

## Supplementary Information

Supplementary Table 1.
