# Supplementary material for: Afidopyropen, a novel insecticide originating from microbial secondary extracts
Source: Sci Rep. 2022 Feb 18;12:2827. doi: 10.1038/s41598-022-06729-z (PMC8857236; doi:10.1038/s41598-022-06729-z)
Supplement: Supplementary file 1 — Supplementary Table 1. [file 41598_2022_6729_MOESM1_ESM.docx]

Supporting Information for:

**Afidopyropen, a novel insecticide originating from microbial secondary extracts**

Ryo Horikoshi^1*^, Kimihiko Goto^1^, Masaaki Mitomi^1^,

Kazuhiko Oyama^1^, Tomoyasu Hirose^2^, Toshiaki Sunazuka^2^ and Satoshi Ōmura^2^

1 Agricultural & Veterinary Research Labs., Agricultural & Veterinary Division, Meiji Seika Pharma Co., Ltd. 760 Morooka-cho, Kohoku-ku, Yokohama, 222-8567, Japan

2 Graduate School of Infection Control Sciences, Ōmura Satoshi Memorial Institute, Kitasato University, Tokyo, Japan

**Supporting Table 1.** Summary for the origin and the stage of insect pests considered in this paper and data observation point after treatment

| pest | origin (Japan) | method | stage/No. of insects a plot | Replication | observation point  (day after treatment) |
| --- | --- | --- | --- | --- | --- |
| *Myzus persicae* | Field populations: Odawara (2002) | Foliar and insect spray with cabbage leaf disk | 1st instar larva/10 | 2 | 6 |
| *Aphis gossypii* | Susceptible^*^and field populations: Aomori (2007), Miyazaki (2011) | foliar to cucumber leaf disk | 1st instar larva/10 | 2 | 6 |
| *Aphis craccivora* | Field  populations:  Odawara (2007) | foliar to fava bean seedling infested with aphids | Mix of all stages  /25 to 50 | 2 | 7 |
| *Trialeurodes vaporariorum* | Field  populations:  Odawara (2002) | foliar to kidney bean leaf disk | Adult/10 | 2 | 6 |
|  |  | foliar to cucumber leaf disk | Egg/  20 to 30 | 2 | 14 |
| *Bemisia tabaci* | Field  populations:  Ibaraki (2007) | foliar to cabbage leaf disks | Adult/10 | 2 | 5 |
| *Pseudococcus comstocki* | Kanagawa (1990s) | foliar to kidney bean leaf disk | 1st instar larva/10 | 2 | 7 |
| *Empoasca onukii* | Field  populations:  Odawara (2007) | foliar to tea shoots | Adult/5 | 2 | 5 |
| *Nilaparvata lugens* | Kagoshima (2000s) | Foliar to rice seedlings | 2nd instar larva/10 | 2 | 7 |
| *Plutella xylostella* | Yokohama (1991) | foliar to cabbage leaf disks | 2nd instar larva/5 | 2 | 6 |
| *Frankliniella occidentalis* | Susceptible^*^ | foliar to kidney bean leaf disk | 1st instar larva/10 | 2 | 6 |
| *Liriomyza trifolii* | Field  populations:  Odawara (2008) | foliar to kidney bean seedling | Adult/7 | 2 | 17 |
| *Oulema oryzae* | Field  populations:  Gotemba (2008) | topical application | Adult/5 | 2 | 2 |
| *Tetranychus urticae* | Susceptible | foliar to kidney bean leaf disk | Egg/  20 to 30 | 2 | 7 |

*Purchased from Sumika Technoservice Corp. (Takarazuka, Japan).
